# Development of a deep learning algorithm for radiographic detection of syndesmotic instability in ankle fractures with intraoperative validation

**DOI:** 10.1038/s41598-025-14604-w

**Published:** 2025-08-14

**Authors:** Joshua Kubach, Tobias Pogarell, Michael Uder, Mario Perl, Marcel Betsch, Mario Pasurka, Stefan Söllner, Rafael Heiss

**Affiliations:** 1 Department of Traumatology and Orthopedics, University Hospital Erlangen, Friedrich-Alexander-Universität Erlangen-Nürnberg, Krankenhausstr.12, 91054 Erlangen, Germany; 2 Institute of Radiology, University Hospital Erlangen, Friedrich-Alexander-Universität Erlangen-Nürnberg, Maximiliansplatz 3, 91054 Erlangen, Germany

**Keywords:** Machine learning, Bone, Computational science, Orthopaedics

## Abstract

Identifying syndesmotic instability in ankle fractures using conventional radiographs is still a major challenge. In this study we trained a convolutional neural network (CNN) to classify the fracture utilizing the AO-classification (AO-44 A/B/C) and to simultaneously detect syndesmosis instability in the conventional radiograph by leveraging the intraoperative stress testing as the gold standard. In this retrospective exploratory study we identified 700 patients with rotational ankle fractures at a university hospital from 2019 to 2024, from whom 1588 digital radiographs were extracted to train, validate, and test a CNN. Radiographs were classified based on the therapy-decisive gold standard of the intraoperative hook-test and the preoperatively determined AO-classification from the surgical report. To perform internal validation and quality control, the algorithm results were visualized using Guided Score Class activation maps (GSCAM).The AO44-classification sensitivity over all subclasses was 91%. Furthermore, the syndesmosis instability could be identified with a sensitivity of 0.84 (95% confidence interval (CI) 0.78, 0.92) and specificity 0.8 (95% CI 0.67, 0.9). Consistent visualization results were obtained from the GSCAMs. The integration of an explainable deep-learning algorithm, trained on an intraoperative gold standard showed a 0.84 sensitivity for syndesmotic stability testing. Thus, providing clinically interpretable outputs, suggesting potential for enhanced preoperative decision-making in complex ankle trauma.

**Supplementary Information:**

The online version contains supplementary material available at 10.1038/s41598-025-14604-w.

## Introduction

Ankle fractures are the most common fracture of a weight-bearing joint, usually occurring in the context of an ankle twisting trauma. Those twisting forces are frequently sustained during sport or falls with disproportionate supination or pronation of the foot^1,2^. Injury patterns range from ligamentous injuries to simple and complex fracture patterns of the medial and lateral malleolus as well as a posterior malleolus. The syndesmosis is a fibrous joint supported by three ligaments (anterior tibiofibular ligament, interosseus tibiofibular ligament, posterior tibiofibular ligament) which can also be affected. Depending on the mechanism of injury the incidence of syndesmosis injury in ankle fractures is between 20 and 100% depending on the fracture pattern^3,4^. Clinically, Squeeze-Test (sensitivity 46%, specificity 79%)^5^, non-weight-bearing dorsal flexion external rotation test (sensitivity 58%, specificity 52%)^5^ and direct pressure pain test (sensitivity 58%, specificity 49%)^5^ in the area of the anterior syndesmosis can be indicative of instability in a fracture setting. Missed syndesmotic instability in fracture patterns can lead to a progressive biomechanical dysfunction, including chronic pain, functional impairment, thus ultimately increasing the risk of post-traumatic osteoarthritis and degenerative joint changes^6^. Early diagnosis and stabilization are critical to prevent long-term complications, as delayed treatment often necessitates complex surgical interventions^3,6^.

Initial radiography remains the first-line imaging modality to diagnose ankle fractures and assess bony displacement suggestive of syndesmotic instability. However, its utility is limited in detecting instability when fracture displacement is subtle or obscured by complex fracture patterns^6^. The native radiological work-up of the syndesmosis instability in ankle fractures achieves a sensitivity of 47–52% and a specificity of 75% even for experienced radiologists and surgeons^7,8^. The lack in sensitivity can be explained by biomechanical studies, as a diastasis > 2 mm could only be consistently achieved with rupture of all 3 syndesmosis ligaments under stress examination^9^. The decisive examination for therapy is performed intraoperatively: After reduction and stabilization of the fractures of the medial, lateral and posterior malleolus, dynamic instability tests are performed using fluoroscopy. The so-called Hook test^10^ or External Rotation Test can be performed^3^. The Hook test appears to be superior to the External Rotation Test, as the External Rotation Test shows significant widening of the medial clearspace in a rupture of the deltoid ligament^11^. The treatment of syndesmosis instability is an additive tri- or quadricortical screw osteosynthesis ranging from the fibula to the tibia around 1,5–3 cm above the talotibial joint space^12^ or utilizing dynamic stabilization like suture button devices^13^. The intraoperative diagnosis that determines the appropriate therapy remains challenging for surgeons, as it is difficult to assess objectively. Furthermore, all potential treatment options must be discussed with the patient prior to surgery. To date, no published studies have specifically investigated the application of CNNs for detecting syndesmotic instability in ankle fractures using preoperative radiographs. Due to the rapid development of diverse types of machine learning algorithms in the last 10 years, there have been increasing applications in medical imaging. In particular, classification, segmentation and detection tasks have been successfully established by algorithms on different medical data. The network architecture used for these tasks are convolutional neural networks (CNN)^14^. CNNs have shown an increased sensitivity and specificity in the detection of different fracture-pattern compared to the imaging evaluation by expert readers alone^15,16^. Systematic approaches for the analysis of radiographic images have been established and will be applied in this study, the protocol used is the CLAIM protocol published by Mongan et al.^17^.

In this study, we present a proof-of-concept to demonstrate the application of a deep learning algorithm in the classification of ankle fractures with the gold standard of intraoperative stability testing of the syndesmosis and simultaneous application of the AO classification. The objective is to develop and validate a deep learning algorithm for the preoperative detection of syndesmotic instability in ankle fractures to enhance diagnostic accuracy of native radiographs.

## Materials and methods

### Dataset

Over the last 5 years (2019–2024) 5412 consecutive patients sustaining ankle fractures could be identified in the clinical information system of our university hospital (Level I trauma center).

Given the retrospective, exploratory design of this study, a priori sample size estimation or statistical power analysis was not conducted. The cohort size was determined by the availability of pre-existing clinical data, with all eligible cases included to maximize analytical breadth while acknowledging potential limitations in generalizability.

1724 of 5412 patients underwent surgical treatment, in 735 a hook test was clearly documented in the operation report with signs of stability or instability. Of the 989 excluded cases, 622 lacked available preoperative radiographs because imaging was performed externally. 110 patients lacked the a.p. (anterior posterior) or lateral view. The ground truth was set as the written documentation of a stable or unstable hook test. In 257 patients a hook test was insufficiently documented in the operation report: Patients without the explicitly stated hook test, even if therapeutic consequences of an unstable syndesmosis like positioning screws were present were excluded. Patients with only radiographic proof of a hook test without explicit mention of stability in the operation report were excluded. Patients with hook testing before full fracture fixation were excluded. 35 patients were excluded due to a direct reduction of the posterior malleolus and therefore potential bias in the traditional variant of the hook test. A total of 10 senior surgeons with subspecialty training in trauma surgery performed the intraoperative hook tests as documented in the operative reports. No retrospective quantitative assessment of the hook tests was conducted.

**Fig. 1:**
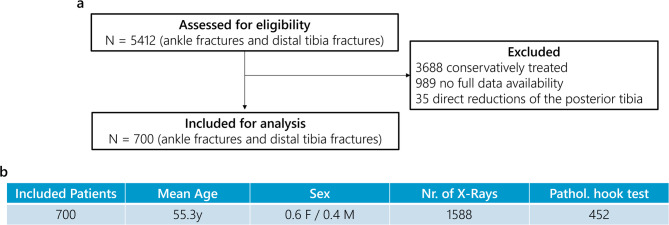
Flowchart of patient inclusion.

Of the 700 included patients, 1588 preoperative radiographs were included in the study evaluation. This mismatch in number of patients in number of radiographs is due to the inclusion of pre- and post-reposition and immobilization imaging. The 1588 digitized radiographs were anonymized and exported as .dicom format from the internal Picture Archiving and Communication System (PACS). Data export and anonymization were conducted via the certified pipeline of the radiology institute, in compliance with the Bavarian Hospital Act (BayKrG) and the General Data Protection Regulation (GDPR), following approval by the local ethics committee (23–173–Br) in May 2023. The study was performed in accordance with the Declaration of Helsinki. The image acquisition protocol included the following parameters: 80% of radiographs were obtained using Agfa systems (models: 3543EZE, DXD30_Wireless) and 20% using Siemens Healthineers systems (model: YSIO X.pree). The average tube voltage was 58.1 kV (range: 51.8–69.8 kV), the average tube current was 466.8 mA, and the average source-to-detector distance was 1150.0 mm. These radiographs were automatically matched with the operative reports to check for the presence of an intraoperative syndesmosis stability check through one investigator. Depending on the intraoperative stability-testing the fractures were assigned to the corresponding groups: syndesmosis stable and syndesmosis unstable. In the same step the AO-classification label was taken out of the surgical report, for later simultaneous classification of syndesmosis stability and AO-classification. The flow chart of the study and the demographic data are shown in Fig. 1 and Table 1.

**Table 1:** Patient demographics and train-validation-test split overview.

Dataset properties	
Mean age	55.3 y
Sex	0.6 F/0.4 M
Cases	700
Training cases	560
Validation cases	70
Test cases	70
Radiographs	1588
Cases with > 2 a.p. & lat. exams	90
Cases including cast/implants	140
Excluded due to image quality	0

**Fig. 2:**
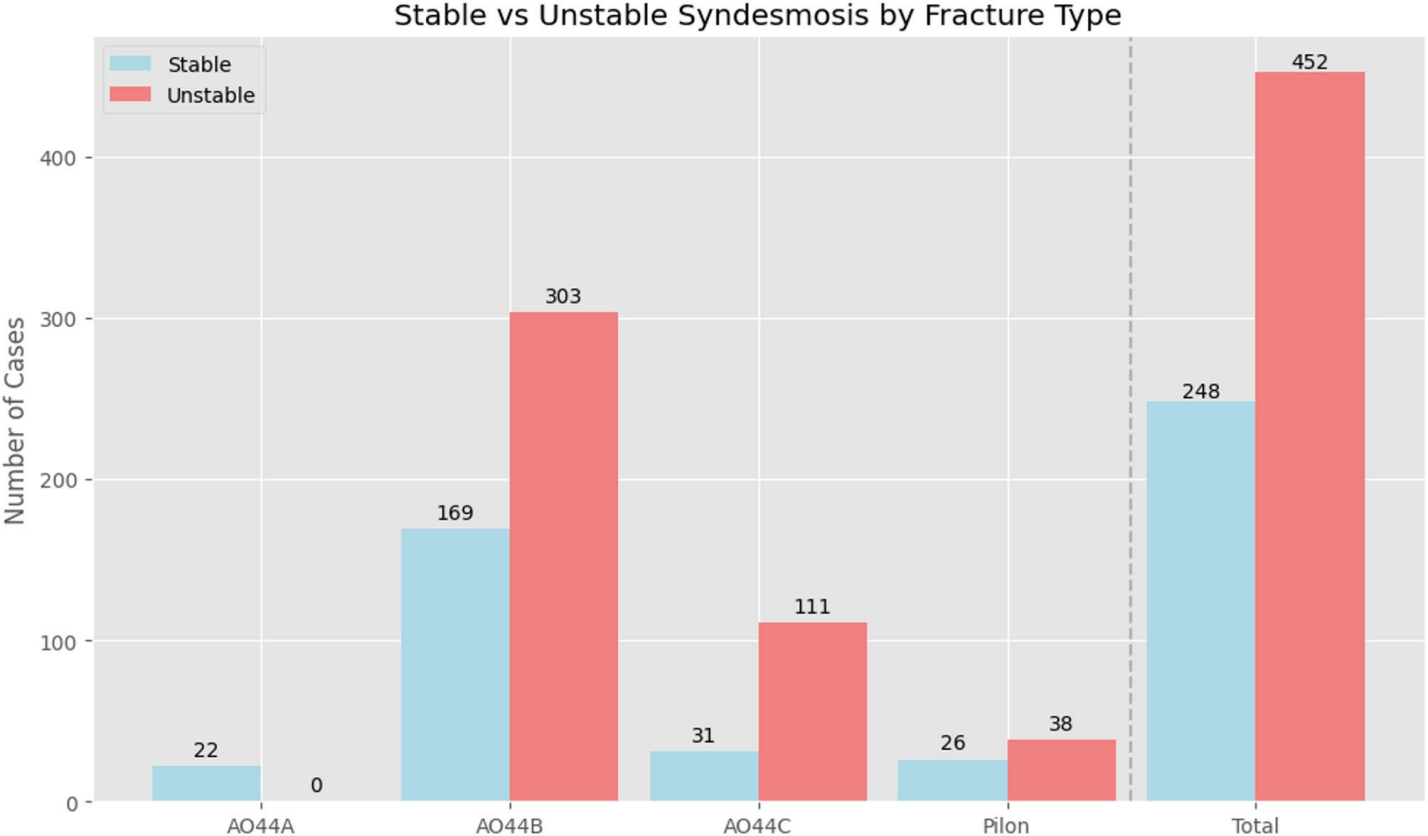
Class distribution of the syndesmosis instability per AO-classified data. Each number represents an individual patient.

The distribution of AO classification results is seen in Fig. 2. In a sub analysis in all AO44A fractures the syndesmosis was deemed stable (100% stable), in AO44B fractures 169 were deemed stable and 303 unstable (35% stable), in AO44C fractures 31 stable and 111 unstable (21% stable), and in Pilon/distal tibia fractures 26 stable and 38 unstable (40% stable). A train-validation-test split of 80–10–10 was utilized as is standard for smaller datasets in most deep learning studies^18^ (Table 1). The partitions were disjointed on patient-level. The data was randomly partitioned based on AO44-subgroup frequency and assigned to Train, Validation and Test-Set by a random number generator utilizing Numpy.

### Deep learning network architecture & data pipeline

**Fig. 3:**
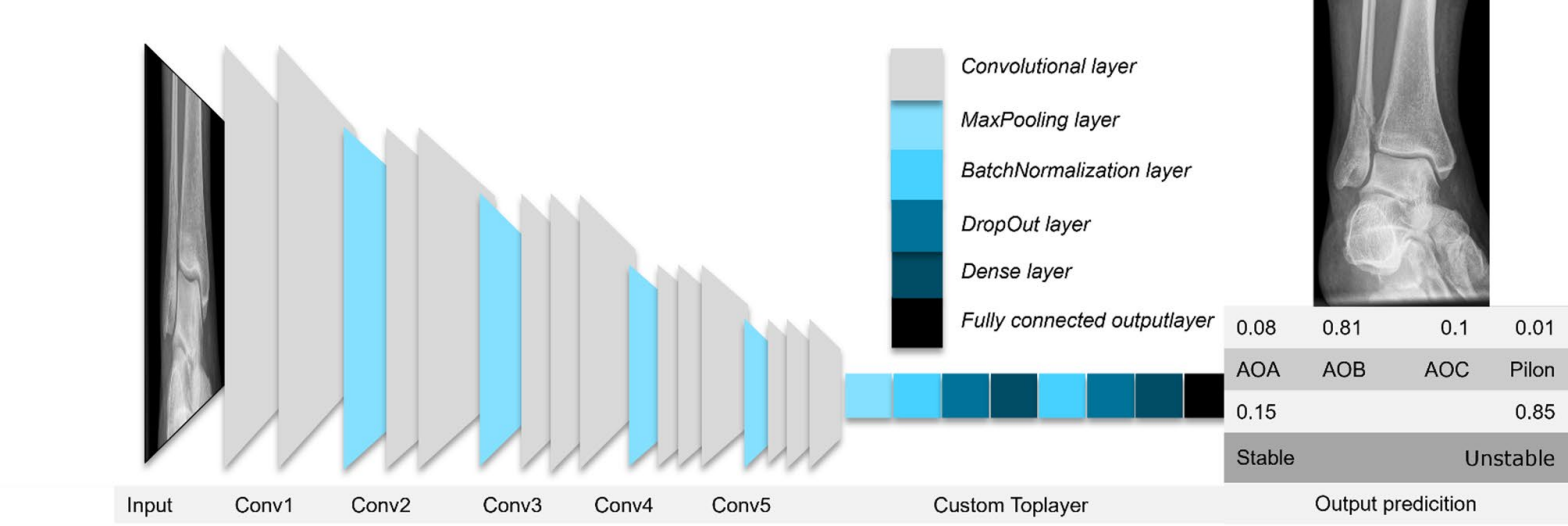
Example network (VGG16) and custom toplayer-configuration.

The implementation of the CNN architecture was done with Python and the open-source package tensorflow2^19^. Various state-of-the-art deep learning networks were applied to the data set and investigated with ImageNet pretrained weights, including VGG16, EfficientNet, NasNetMobile, ResNet^20–22^. In the initial phase of model development, we systematically trained and evaluated several established convolutional neural network architectures on a randomly generated subset of our dataset to identify the architecture that achieved the highest diagnostic performance for our specific classification task. The basic network architecture was retained and the toplayers (BatchNormalization^23^, DropOut, MaxPooling) of were adapted to minimize overfitting in accordance with current recommendations (Fig. 3)^17^. The model is initialized with a transfer learning approach utilizing the ImageNet pre-trained weights^24^. To retain pretrained features during transfer learning Network Freezing was utilized during early training stages only training the custom toplayer^24^.

Image preparation is central to any deep learning network application. Each image was normalized and augmented using a randomized combination of geometric transformations (shear, elastic), texture/edge modifications (blur, sharpen, emboss, edge detect), stochastic perturbations (dropout), and color distortions (contrast, brightness, hue permutation), applied either globally or to localized regions. The application was done in a custom image generator, so that no additional storage of the images was necessary, and the training efficiency was maximized by random augmentations at each training epoch. An additional improvement in domain adaptation and robustness of the network was demonstrated in informatic studies^17^. To battle class imbalance which is present in our dataset as seen in Fig. 2 we implement weighted classes to adjust the loss function to assign higher weights to underrepresented classes, forcing the model to prioritize their correct classification and balance their influence during training.

The training was applied with the following optimizations: Cyclic Learning Rates (CLR)^25^ dynamically adjust learning rates to escape local minima, as demonstrated in epilepsy histopathology classification where CLR (1e^−7^ to 1e^−3^) stabilized training and cross-validation accuracy^26^. Early Stopping was applied if the validation loss did not improve for 10 epochs^27^, thus preventing overfitting by halting training when validation metrics meet a plateau. Training was performed with a batch size of 128 and the Adam Optimizer.

### Performance evaluation

To validate model performance the dataset was partitioned into 10 stratified folds balancing the baseline syndesmotic instability across the subsets. For each iteration one fold was reserved as the validation set, while the others were utilized as the training set was used utilizing a random assignment of cases to each fold. Performance metrics included accuracy, loss, sensitivity, specificity, precision and F1 score.

To evaluate the algorithms performance on the test set, an unseen test set was held back and only normalized as preprocessing. The test set assembled 70 randomly picked cases, which were not connected to the training and validation set. The randomization was obtained through Numpy random number generation balanced across all AO-subclasses. The test set consisted of 2 AO44-A cases, 48 AO44-B cases, 14 AO44-C cases and 6 Pilon cases. Then a.p. (anterior posterior) and lateral images of each case were predicted and a mean probability was computed (p = (p^ap^ + p^lat^)/2) with a > 0.5 threshold for a prediction on the individual patient. The classification results were evaluated by accuracy, sensitivity, specificity, precision and F1 Score as well as confidence intervals.

### Visualization

Guided Score-weighted Class Activation Mapping (GSCAM) enhances diagnostic explainability in syndesmosis instability assessment by integrating CLIP-derived attention mechanisms with guided backpropagation to pinpoint clinically critical imaging biomarkers^28^. Unlike gradient-dependent methods like Grad-CAM^29^, this technique leverages confidence scores from target class activations to generate spatial attention maps, reducing noise while preserving high-resolution anatomical details crucial for detecting those subtle ligamentous injuries.

### Hardware

We implemented our approach on a local server running Ubuntu with one NVIDIA GeForce GTX 3080 Ti, Intel CPU i7 12700, 64 Gb RAM, CUDA 11.7, and cuDNN 8.0.

## Results

### Pretesting and k-fold crossvalidation results

Initially, different network architectures were trained on 50% of a random portion of the main dataset. In the subset, NasNetMobile was found to be the best model for the given dataset on a small validation and testing dataset (Supplement 1). Next, the data pipeline was tested with different augmentation strengths. Through an iterative process on our in-house dataset a medium amount of noise through data-augmentation seemed to obtain the best results.

**Fig. 4:**
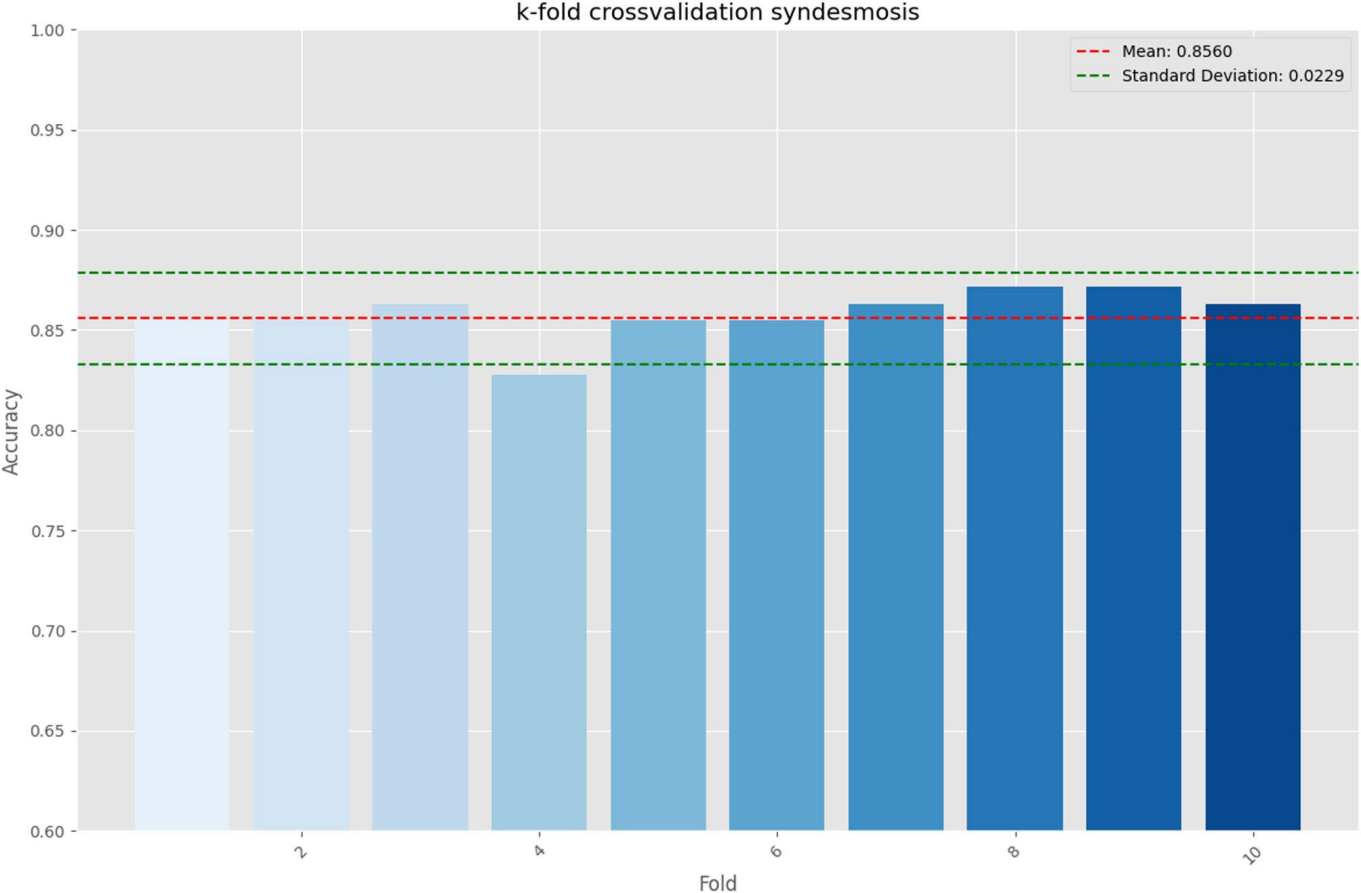
K-fold cross validation results showing homogeneous accuracy, except for fold 4.

This model was then trained on the whole dataset and k-fold crossvalidation was applied, where a mean accuracy of 0.8560 with a standard deviation of 0.0229 was achieved in the crossvalidation process (Fig. 4). The variation of accuracy in fold 4 is noted due to comparatively many pilon fractures/distal tibial shaft fractures in validation subset 4.

### Syndesmosis stability testing

**Fig. 5:**
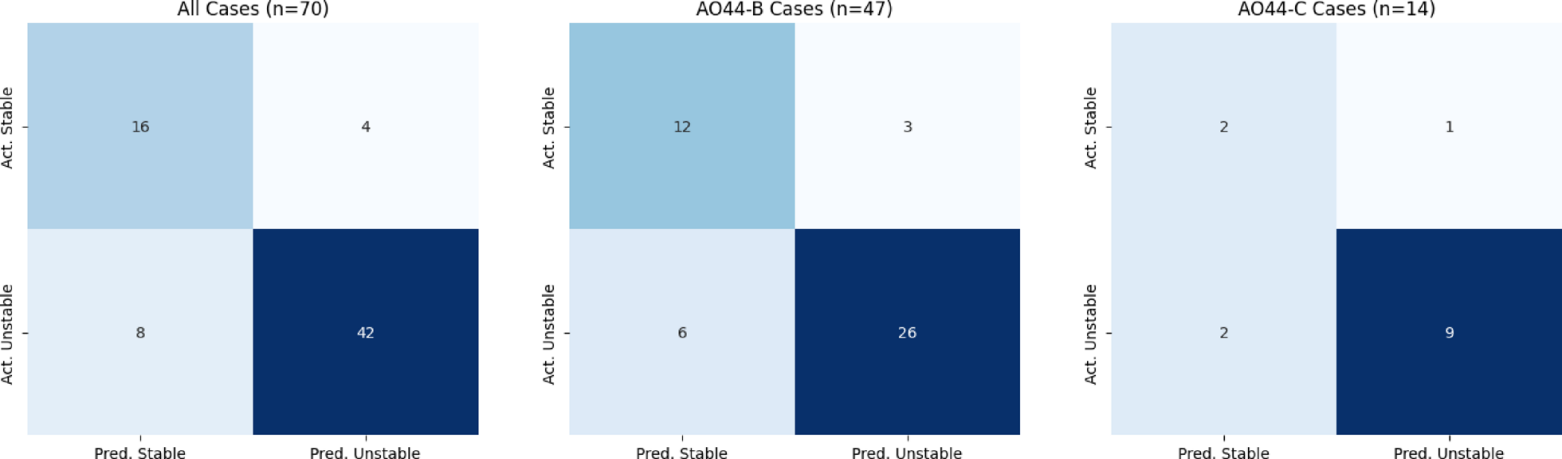
Confusion Matrices for the average syndesmosis instability classification as well as AO44-B & AO44-C subset classification.

Based on the aforementioned findings, the final model was determined using the best performing model in the tenfold cross validation. The previously unseen, randomly generated and AO-subgroup stratified test set was predicted using this model. On the unseen test-set scoring an overall sensitivity of 0.84 with a Wilson Confidence Interval (CI) of 0.71 to 0.92, an overall specificity of 0.81 (95% CI 0.58 to 0.9), an overall precision of 0.91 and an overall F1-Score of 0.87 (Table 2). The performance overview as well as the confusion matrix for the syndesmosis instability prediction is shown in Fig. 5.

**Table 2:** Performance metrics for the average syndesmotic stability classification as well as for the AO44-B and AO44-C subgroups.

	Overall performance	AO44-B	AO44-C
Metrics	Values [95% CI]	Values [95% CI]	Values [95% CI]
Sensitivity	0.84 [0.71, 0.92]	0.81 [0.65, 0.91]	0.82 [0.52, 0.95]
Specificity	0.8 [0.58, 0.92]	0.8 [0.55, 0.93]	0.67 [0.21, 0.94]
Precision	0.91	0.9	0.9
F1 Score	0.97	0.85	0.86

The AO44-B cohort achieved a sensitivity of 0.81, specificity of 0.80, precision of 0.90, and F1 score of 0.85. For the AO44-C cohort, sensitivity was 0.82, specificity 0.67, precision 0.90, and F1 score 0.86.

All fractures in the AO44-A subcohort were correctly classified as stable. For the Pilon/distal tibia subset (n = 6), sensitivity and specificity were 0.75 and 0.50, respectively (Fig. 5).

### AO44-classification testing

The multiclass algorithm achieved overall sensitivity of 0.91 (95% CI 0.81–0.96), specificity of 0.96 (0.93–0.98), precision of 0.91, and F1-score of 0.91 for AO-44 classification and distal tibia/pilon fracture categorization. Subgroup analyses demonstrated:

- *AO44-B* Sensitivity 0.95/Specificity 0.86
- *AO44-C* Sensitivity 0.86/Specificity 0.96
- *Pilon/Distal Tibia* Sensitivity 0.83/Specificity 0.98
- *AO44-A* Sensitivity 0.50/Specificity 0.98 (*n* = *2*), reflecting limited interpretability due to minimal samples.

High specificity (> 0.95) across all subgroups underscores reliable detection of subtypes.

### CNN visualization

**Fig. 6:**
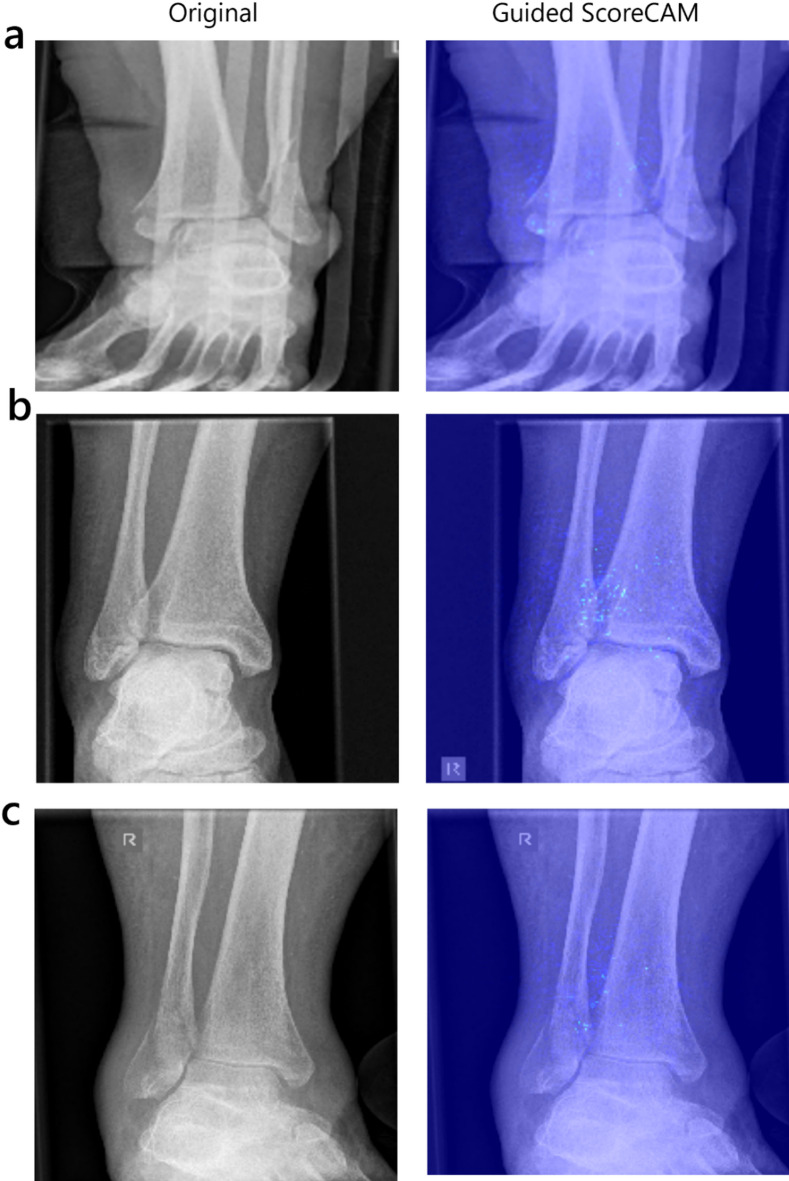
(**a**): Guided Score-CAM in higher grade fracture dislocation in an AO 44 B2 fracture of a 58 y old female with an unstable syndesmosis. (**b**) AO 44B1 fracture in a 40 y old female depicting an unstable syndesmosis with minimal radiologic signs in the a.p. x-ray. (**c**) False positively predicting an AO44 B1 Fracture in a 67 year-old male with Guided Score CAM focusing on the syndesmotic region.

To further classify the accuracy of the model, each test case was visualized with different Visualization techniques with Guided ScoreCAMs performing the most precise. Overall, it was possible to recognize from the sum of the evaluated image data that, according to Supplement 2 and Fig. 6, a comprehensible decision-making process had taken place through the generated heat maps. Interestingly, the position of the medial malleolus played an important decision–making factor in the role of determining the syndesmotic stability, being a recurring theme, in higher grade fracture dislocation, as seen in Fig. 6a. The medial malleolus, when moving in conjunction with the talus, was focused on a lateral shift dislocation type fracture. Thus, indicating an understanding of pathobiomechanics as the deltoid ligament is intact maintaining the relationship to the talus and moving in conjunction. The tibiofibular overlap loss reflects the fibulas lateral displacement from the tibial incisura and is indicative of syndesmotic instability^30^

.

Furthermore, in Fig. 6b a minimally displaced AO44B1 fracture presented with an interoperative syndesmosis instability which is not apparent on the initial x-ray. The algorithm classified the syndesmosis as unstable and focused on the syndesmotic region. Interestingly also a case depicted in Fig. 6c was observed where the algorithm classified the syndesmosis as unstable contrary to the surgical report, while focusing on the syndesmotic region.

## Discussion

In the present study we developed a deep learning algorithm that can detect syndesmosis instability with a sensitivity of 0.84 and a specificity of 0.8 as well as classify AO-44-classification on x-rays in the preoperative work-up of ankle fractures. Furthermore, the results were visualized for traceability and quality control. As far as we know, this is the first study to systematically apply a deep-learning algorithm for the detection of syndesmotic instability. The following goals were applied to our machine learning algorithm:

1. Building a multiclass algorithm to obtain a higher pretest probability for the operative planning for operative candidate ankle fractures.
2. Translationally utilizing the current gold standard of the intraoperative hook-test for the deep-learning aided analysis of pretherapeutic X-rays.
3. Traceability and visualization of the decision-making process.

In syndesmosis instability diagnosing our algorithm reached an overall sensitivity of 0.84 with a 0.81 specificity. This optimizes the probability of a correct diagnosis, based on X-ray findings alone as well as pretest probability to detect a syndesmotic instability before an operation. The surgeon can discuss additional intraoperative steps with impact on the operation and aftercare before the operation.

The number of patients in the different AO-subclassifications is comparable to other research with the exception of AO-44A, because the strictly operative cohort as our inclusion criteria^18^. In the AO44-B subcohort a syndesmotic instability was found in 64% of all cases 303/472. This is higher than reported in a recent systematic review^31^ and historic data^32,33^. Lim et al. focused on Weber B fracture patterns without additional medial malleolus or posterior malleolus fractures as characterized by the AO44 B2 and B3 subclassifications, thus explaining the increased rate of syndesmotic instabilities in our cohort^31^. In the AO44-C subcohort a syndesmotic instability was present in 78% (111/141 cases) which is in line with recent studies describing instability rates of up to 75%^31^. Our results show an outperformance of guessing and published data of relevant syndesmotic instability in initial x-ray workup^6–8^.

Our results for syndesmotic instability detection outperform the assessment of the syndesmosis in conventional reading of ankle radiographs with a sensitivity of 47% and a specificity of 100% in an MRI controlled study utilizing the AO-classification with additional radiographic measurements (tibiofibular overlap, tibiofibular clearspace, medial clearspace.)^34^. These measurements seem to vary by specimen and rotation as shown in a cadaveric study^35^. In another study, an operatively treated Weber B cohort with MRI and arthroscopic control demonstrated a sensitivity of 52% and specificity of 100% with conventional radiologic assessment alone^8^. Overall, there is no definitive agreement on which factors are certain to constitute syndesmosis instability in conventional radiologic assessment. Complementary computed tomographic (CT) imaging for more complex ankle fractures can be added for preoperative planning and fracture classification. Indirect radiographic signs-such as subluxation patterns in the distal tibio-fibular joint, direct measurements of tibiofibular clear space widening and fibula rotation, and specific avulsion or fracture patterns-can help predict syndesmotic instability, with reported sensitivity of 74–82% and specificity of 75–100%^7,8,36^. Weightbearing CT Scans and especially automated 3D analysis of CT scans showed promising results but are not standard care^37,38^. An addition to the diagnostic algorithm for ankle fractures is MRI. Of the discussed imaging modalities, it is the only imaging technique that can directly visualize the syndesmosis as a ligamentous structure. A sensitivity of 91–100% and specificity of 85–100% can be achieved^7,8^. However, the non-ubiquitous availability of CT and MRI and the costs are problematic.

As shown in previous research, deep learning seems to be a good tool for classifying ankle fractures based on AO classification as already shown by Olczak et al.^18^. In this study a randomly initialized ResNet was utilized to predict a test set of 400 unique patients reaching an overall AUC of 0.90. In another application of a deep learning algorithm, only the fracture itself was detected in different X-ray planes, where a sensitivity of 98.7% and a specificity of 98.6% could be achieved^15^. Applying our CNN, we could show a sensitivity of 0.91 and a specificity of 0.96 for the overall AO-classification of the fracture. Interestingly the algorithm performed worst in the AO44–A subgroup, although it contained only 2 patients due to the already discussed strictly operative cohort. These results mirrored the results of the AO44–A subgroup analysis of Olczak where the AO44–A subgroup also performed the worst^18^. AO44–A fractures represent infrasyndesmotic fibular fractures that typically have lower rates of syndesmotic injury (approximately 7.14%) compared to AO44–B (31.15%) and AO44-C (88.50%) fractures^39^. This epidemiological reality, combined with our limited sample size and operative cohort, creates a class imbalance that may affect model training and validation.

The intraoperative hook test served as the reference standard for syndesmotic instability assessment, consistent with its established role in guiding therapeutic decisions^8,9,31^. However, its diagnostic reliability is influenced by interobserver variability and heterogeneous sensitivity estimates across studies^11,31,40^. Thus, limiting our reference standard as not retrospective standardization or quantification is possible due to our study design. Direct posterior malleolus fixation enhances syndesmotic stability, potentially skewing intraoperative instability assessments. Our study excluded 35 such cases to isolate confounding biomechanical effects^41,42^.

Another crucial point of discussion is the need for external validation. Utilizing external data from different X-ray machines and surgical teams-each potentially applying slightly different standards for hook testing the syndesmosis after fixation-is essential for developing a robust deep-learning algorithm. In studies with small cohorts, overfitting remains a significant concern. To address this, we implemented an extensive preprocessing and regularization pipeline that included normalization, batch normalization, dropout, cyclic learning rates, class weighting, the use of medium-sized models, and quality control through visualization with various class activation maps.

Looking ahead, we recognize the importance of multicenter collaboration for the further development of algorithms aimed at detecting ankle sprains and fractures. Such collaboration is essential to address domain adaptation challenges that arise from variations in imaging equipment, acquisition protocols, intraoperative testing standards, and patient demographics across different institutions. In our future work, we plan to establish a comprehensive, multimodal dataset with contributions from multiple centers. This dataset will include preoperative 3D imaging modalities such as CT and MRI, as well as intraoperative arthroscopic assessments using both standard and needle arthroscopy, in order to provide an optimal reference standard for detecting syndesmotic instability^8^. Through multicenter external validation, we aim to enhance the robustness, generalizability, and clinical applicability of our algorithms across diverse healthcare settings. This would be promising not only in fracture care but also in isolated syndesmotic ruptures and ligamentous injuries of sprained ankle patients. A model could be implemented into routine work-up of radiographic analysis of an ankle radiographs to assist the traumatologist and radiologist in initiating important diagnostics earlier. Multiple prospective multicenter studies regarding validation through multimodal approaches are needed to begin tackling regulatory hurdles critical for deployment.

Our study has several limitations. The current investigation employs a single-center, retrospective design that lacks external validation datasets, which restricts the transferability and broader applicability of our findings. This absence of external validation constitutes a critical limitation, as multicenter validation across diverse clinical environments and patient populations is essential for confirming both the generalizability and robustness of deep learning algorithms in clinical practice.

Due to the operative nature of our inclusion criteria, our study is limited by a relatively small dataset and notable class imbalance:The small sample size of the AO44-A subgroup (test-set n = 2) prevents meaningful statistical analysis of model performance in AO44-A cases and may contribute to the observed underperformance in this subgroup. Furthermore, due to retrospective nature missing data is unavoidable leading to potential bias regarding generalization. Intraoperative hook test variability among 10 surgeons, combined with the retrospective design’s inability to standardize methodology or assess interobserver agreement, may have affected our reference standard reliability. The hook test is mainly testing for coronar instability and may not fully capture sagittal or rotational instability.

## Conclusion

We present a proof-of-concept for a deep learning algorithm that integrates intraoperative findings with early radiographic diagnostics, improving the preoperative detection of syndesmotic instability in ankle fractures and supporting timely, accurate surgical decision-making to enhance patient outcomes.

## Supplementary Information

Below is the link to the electronic supplementary material.

Supplementary Material 1

## Data Availability

The datasets generated during and analyzed during the study are not publicly available due to the protection of personal information and limitation of ethical approvement but are available from the corresponding author on reasonable request.
