# Supplementary material for: Development of a deep learning algorithm for radiographic detection of syndesmotic instability in ankle fractures with intraoperative validation
Source: Sci Rep. 2025 Aug 14;15:29880. doi: 10.1038/s41598-025-14604-w (PMC12354714; doi:10.1038/s41598-025-14604-w)
Supplement: Supplementary file 1 — Supplementary Material 1 [file 41598_2025_14604_MOESM1_ESM.docx]

#
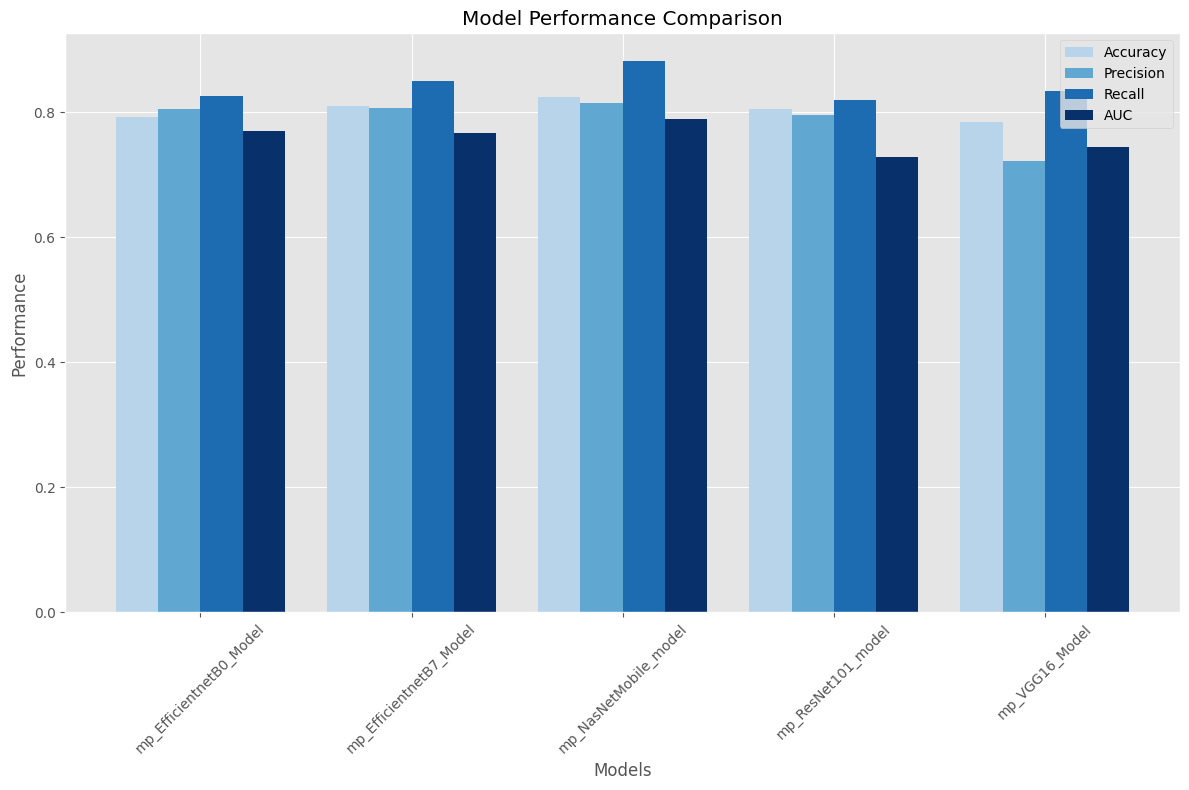
Supplements

*Supplement 1 | Different state-of-the-art models performance comparison on a 50% subset.*

*
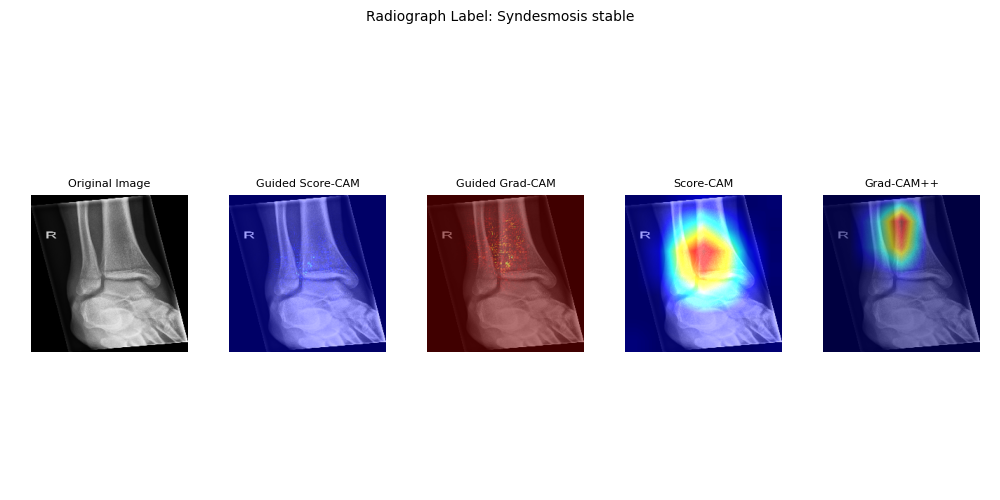
 Supplement 2 | Overview of different visualization techniques in the syndesmotic instability classification. This case represents an a.p. radiograph of a 15y male with undislocated Weber B fracture and a stable syndesmosis.*
